# Graphics processing units-accelerated adaptive nonlocal means filter for denoising three-dimensional Monte Carlo photon transport simulations

**DOI:** 10.1117/1.JBO.23.12.121618

**Published:** 2018-11-29

**Authors:** Yaoshen Yuan, Leiming Yu, Zafer Doğan, Qianqian Fang

**Affiliations:** a Northeastern University, Department of Electrical and Computer Engineering, Boston, Massachusetts, United States; b Northeastern University, Department of Bioengineering, Boston, Massachusetts, United States; c Harvard University, John A. Paulson School of Engineering and Applied Sciences, Cambridge, Massachusetts, United States

**Keywords:** Monte Carlo, image denoising, photon migration, light propagation, image processing

## Abstract

The Monte Carlo (MC) method is widely recognized as the gold standard for modeling light propagation inside turbid media. Due to the stochastic nature of this method, MC simulations suffer from inherent stochastic noise. Launching large numbers of photons can reduce noise but results in significantly greater computation times, even with graphics processing units (GPU)-based acceleration. We develop a GPU-accelerated adaptive nonlocal means (ANLM) filter to denoise MC simulation outputs. This filter can effectively suppress the spatially varying stochastic noise present in low-photon MC simulations and improve the image signal-to-noise ratio (SNR) by over 5 dB. This is equivalent to the SNR improvement of running nearly 3.5× more photons. We validate this denoising approach using both homogeneous and heterogeneous domains at various photon counts. The ability to preserve rapid optical fluence changes is also demonstrated using domains with inclusions. We demonstrate that this GPU-ANLM filter can shorten simulation runtimes in most photon counts and domain settings even combined with our highly accelerated GPU MC simulations. We also compare this GPU-ANLM filter with the CPU version and report a threefold to fourfold speedup. The developed GPU-ANLM filter not only can enhance three-dimensional MC photon simulation results but also be a valuable tool for noise reduction in other volumetric images such as MRI and CT scans.

## 1 Introduction

The development of innovative biophotonics techniques relies on accurate and efficient photon propagation models, especially when imaging complex human anatomy. The importance of developing fast and accurate light propagation algorithms in general media is further highlighted by the increasing utility of model-based methods in optical image acquisition and image processing. The radiative transport equation (RTE) most realistically describes the light propagation in a general random media, such as human tissues. Nevertheless, directly solving the RTE is computationally expensive and memory intensive due to the high dimensionality of the solution. On the other hand, the diffusion equation (DE) provides a good approximation to the RTE in a scattering-dominant media^1^^,^^2^ and can be computed efficiently using finite-element (FE)-based numerical solvers.^3^^,^^4^ However, it has been shown that solving the DE in regions that contain low-scattering media, such as cerebral spinal fluid (CSF) in the brain and other void-like regions, can lead to erroneous solutions.^5^^,^^6^

Unlike other RTE solvers that rely on variational principles, the Monte Carlo (MC) method is a stochastic solver that runs a large number of independent random trials of photon packets to obtain light intensity distributions.^7^ Although the steps needed for simulating a single-photon random movement are relatively simple to implement, tens of millions of photons are often needed to obtain results of sufficient quality, taking up to several hours computing time when a traditional serial MC algorithm is used.^8^ To improve the computational efficiency, a number of hybrid models have been studied over the years, combining DE-based solutions for diffusive regions with MC-solutions for low-scattering regions.^9^^,^^10^

Over the past decade, the rapid advancements in graphics processing units (GPU) have offered a new opportunity to accelerate MC simulations. Massively parallel MC algorithms have been proposed for simple homogeneous,^11^ layered,^12^ and three-dimensional (3-D) heterogeneous media.^13^^–^^15^ Due to the independence between random photons, GPU-based MC algorithms have demonstrated significant speed improvement, ranging from a few hundred fold to over thousand fold, when compared with serial MC modeling.^11^^,^^13^^,^^15^ This has shortened the MC runtime from hours to seconds. Despite this dramatic improvement in speed, the desires to simulate an even larger number of photons in extended, heterogeneous volumes and to model arrays of sources and detectors in tomographic settings continue to motivate researchers toward further reduction of MC runtimes. While it is always feasible to reduce the MC modeling time by simulating less photons, stochastic noise can become dominant in those low-photon simulations, resulting in loss of accuracy.

To reduce the intrinsic stochastic noise without running a large number of photons, applying signal processing techniques to “denoise” a low-photon MC solution has been investigated in limited areas of research such as radiation dosage estimation^16^^–^^20^ and, more recently, computer graphics rendering.^21^^–^^24^ Conventional MC denoising techniques have primarily focused on removing global noise.^21^ Only in recent years, noise adaptive filters, considering the spatially dependent noise models, have been proposed.^21^^,^^25^ Nonetheless, denoising MC simulations of electron beams or ionizing photon beams in the context of radiation dose calculations typically involve more complicated filter designs and are computationally demanding;^19^ the efficacy of these filters varies substantially depending on both anatomy and noise level.^19^ The recent progress in MC-denoising in computer graphics focuses on machine-learning (ML)-based denoising techniques;^24^^,^^26^ however, these methods were largely designed for denoising two-dimensional (2-D) low bit-depth image data. In comparison, MC photon simulations in diffusive media typically produce fluence maps that are smooth with high dynamic range and spatially varying noise, typically represented by floating-point numbers. These major differences between image and noise characteristics render most of the existing ML-based denoisers unsuited for processing fluence images without modifications. As far as we know, there are no reported studies on denoising images produced from low-energy photon transport MC simulations. The noise models of the MC photon simulation outputs, such as fluence and partial path-lengths,^8^ are generally known to be complex and not well understood.^27^ However, it has been generally agreed that the dominant form of noise in the fluence images after simulating many individual photons or photon packets^7^ follows a scaled Poisson^27^^,^^28^ or Gaussian^29^ distribution.

It is our opinion that an ideal MC denoising filter should possess the following characteristics: (C1) it must be effective in removing the noise of the expected MC noise distributions (Poisson or Gaussian), (C2) it must be adaptive to spatially varying noise, (C3) it should not remove sharp features from the underlying images or introduce a systematic bias, and (C4) it must have good computational speed so that it is faster to achieve the desired image quality than running more photons. Properties C1 and C2 in the above wish-list are related to the fact that Poisson or shot-noise^28^ is the dominant noise form in MC photon transport simulations. In the regions traversed by more than a few dozens of photons, the noise is well approximated by the Gaussian distribution; in the low-photon regions, the noise has a Poisson distribution. The shot-noise is also known to be intensity-dependent as the standard-deviation of the noise at a given spatial location is equal to the square-root of the number of photons traversing through it.^30^^,^^31^ Therefore, spatial adaptivity is crucial. Property C3 is important to preserve the high contrast profiles next to a point source and the fluence discontinuity across the boundary of an inclusion with a refractive index contrast. Property C4 is currently quite challenging to achieve, especially given the drastically accelerated MC simulation speeds achieved over the past decade using GPUs.

Among common filters proposed for 3-D image denoising, simple Gaussian filters are fast to compute (C4), effective for high-photon regions (C1), but do not have spatial adaptiveness (C2) or preserve sharp edges (C3). Gaussian filters combined with the Anscombe transform (AT) extend effectiveness to the low-photon regions but still limited in adaptiveness and edge-preservation. The nonlocal means (NLM) filter^30^ was shown to be highly effective in filtering Gaussian-type noise (C1) with the additional benefit of excellent edge preservation (C3). In recent years, an adaptive NLM (ANLM) filter was proposed^32^^–^^34^ for processing MRI images with adaptive noise (C2). Similar characteristics were found in the recently developed block-matching and four-dimensional filtering (BM4D) algorithm.^35^^,^^36^ However, the slow computation speeds (C4) in the ANLM and BM4D filters restrict their use, especially when processing GPU-accelerated MC simulations.^35^

GPU-accelerated 3-D adaptive filters can potentially bring excellent computational efficiency (C4) to the state-of-the-art 3-D filters and make them suitable for denoising GPU MC simulations. Granata et al.^37^ reported significant speed improvements using a GPU-based ANLM filter. However, a number of simplifications were found when comparing it with the original ANLM filter,^34^ including removal of the preselection of nonlocal patches, 2-D instead of 3-D estimation of noise variance,^34^^,^^38^ and reduced data precision (2-byte integers for MRI data). For a typically sized volume, the filtering speed requires further improvement in order for it to be useful in most typical MC simulations ($10^{6}$ to $10^{8}$ photons). Although the GPU-BM3D filters^39^^,^^40^ reported excellent speed, they are designed for filtering two-dimensional (2-D) images and are not suited for 3-D denoising. As far as we know, there is no publication on GPU-BM4D filters. Developing a more general GPU-based 3-D noise-adaptive filter with higher working precision and efficiency could benefit a wide variety of medical image data processing tasks, including improving 3-D MC simulation outcomes.

In this work, we describe a significantly improved GPU-accelerated ANLM filter and study its applications in denoising 3-D MC photon transport simulation images. The new filter shows a twofold to threefold speed improvement from the state-of-art GPU implementations and can work with higher data precisions. We have also systematically quantified the image quality improvement in denoising MC generated image data. We show that the denoising step can generate an average 5-dB SNR improvement; this is equivalent to the result of running 3- to 3.5-fold more photons. The robustness of the proposed methods is demonstrated using 3-D simulations from various photon numbers in both homogeneous and heterogeneous domains.

The remainder of this paper is organized as below. In Sec. 2, we present the basic formulation of the ANLM filter and the details of our improved 3-D GPU ANLM filter. The procedures to apply the proposed filter to enhance MC image quality and evaluation metrics are also described. In Sec. 3, we compare the filtered and unfiltered MC simulations, including results from both homogeneous and heterogeneous domains at various photons numbers, and quantify the improvement using the developed metrics. In addition, we also compare the computation time of the ANLM filtering using GPU versus CPU. Overall runtimes combining GPU-based MC simulations with GPU-based ANLM filters are calculated and discussed for three benchmark problems. In Sec. 4, we summarize our findings and describe future research plans.

## 2 Method

### 2.1 Adaptive Nonlocal Means Filters and Feature Comparisons

The original CPU-based ANLM filter^34^ contains a number of key features, such as calculation of the weighted averages of nonlocal patches, preselection of nonlocal patches^38^ for better image quality, spatial noise adaptivity, and wavelet sub-band mixing.^38^^,^^41^ A comparison between the CPU-based ANLM,^34^ the previously published GPU-ANLM filter,^37^ and the GPU-ANLM filter developed in this work is shown in Table 1.

**Table 1:** A feature-matrix comparison between the published and proposed ANLM filters. The algorithm-related features are explained in Sec. 2.1. The GPU-related features are explained in Sec. 2.2.

Main features	CPU-ANLM^34^	GPU-ANLM^37^	This work
Compute type	CPU	GPU	GPU
Data type	Double (8-byte)	Short integer (2-byte)	Float (4-byte)
Block-wise update	Yes	No	No
Nonlocal patch preselection	Yes	No	Yes
Adaptive noise $\sigma^{2}$ estimation	3-D	2-D	3-D
Filtering Gaussian noise	Yes	Yes	Yes
Filtering Rician noise	Yes	Yes	Yes
Wavelet sub-band mixing	Yes	No	Yes
GPU block configuration	—	16×16×1	8×8×8
GPU texture memory	—	No	Yes
Source code	Open-source	Closed-source	Open-source

The algorithm details listed in Table 1 can be found in Ref. 34. Briefly, in a 3-D image, the value of any voxel located at $x_{i}$ in the filtered image, $u^{\prime }(x_{i})$, is determined by the weighted average of all the voxels, $x_{j}$, in a 3-D cubic search volume $V_{i}$ in the neighborhood of $x_{i}$, i.e.,

$$u^{\prime }(x_{i})=\underset{x_{j}\in V_{i}}{\sum }w(x_{i},x_{j})u(x_{j}),$$ (1)

where $w(x_{i},x_{j})$ is the weight for voxel $x_{j}$ and is calculated using two small cubic volumes, one centered at $x_{i}$, referred to as the “local patch” $P_{i}$, and the other one centered at $x_{j}$, referred to as the “non-local patch” $P_{j}$; the local and nonlocal patches have the same size, which is smaller than the size of $V_{i}$. The weight can then be expressed as

$$w(x_{i},x_{j})=\frac{1}{Z_{i}}\exp[-\frac{{\left\|u(P_{i})-u(P_{j})\right\|}_{2}^{2}}{h{\left(x_{i}\right)}^{2}}],$$ (2)

where ${\left\|u(P_{i})-u(P_{j})\right\|}_{2}$ denotes the Euclidean ($L_{2}$) distance between the local and nonlocal patches, $Z_{i}$ is a normalization factor to ensure $\sum_{x_{j}\in V_{i}}w(x_{i},x_{j})=1$, and $h(x_{i})$ is a spatially adaptive parameter that regularizes the strength of the filter; $h(x_{i})$ is estimated using the standard deviation of the image volume adjacent to the center voxel,^34^ i.e., $\sigma (x_{i})$, which is in turn approximated as

$$\sigma^{2}(x_{i})=\min_{x_{j}\in V_{i}}[R(x_{i}),R(x_{j})],$$ (3)

where $R(x)=u(x)-\overset{¯}{u(B_{x})}$ is the residual, i.e., the difference between the raw input u(x) and its box-filtered version, $\overset{¯}{u(B_{x})}=\underset{x_{k}\in B_{x}}{\sum }u(x_{k})/N_{B}$, $B_{x}$ is a cubic domain centered at x, and $N_{B}$ is the number of voxels in $B_{x}$. For simplicity, the search volume $V_{i}$, local/nonlocal patch $P_{i}/P_{j}$, and the box-filter region $B_{x}$ are considered to be cubic domains, with radii $r_{V}$, $r_{P}$, and $r_{B}$, respectively. For example, the search volume $V_{i}$ is comprised of ${\left(2r_{V}+1\right)}^{3}$ voxels, and so on.

A few modifications to the above algorithm were introduced by Manjón et al.^34^ and Coupé et al.^38^ First, the “block-wise” implementation updates all voxels in the local-patch instead of only the center voxel as shown in Eq. (1). While this method allows skipping of every other voxel, it requires data exchange between different voxels within the local patch, which makes it difficult to implement in the GPU. In both Ref. 37 and this work, the voxel-based update scheme is used. Second, the nonlocal patches with low similarities to the local patch can be ignored, referred to as “preselection,”^34^ to improve image quality and accelerate computation. Preselection is not considered in the previously published GPU-ANLM work^37^ but is fully implemented in this work. Third, a wavelet transformation combining two different patch sizes can be used to improve filtering performance.^41^ Previously, the two filtering steps were performed sequentially on the CPU.^34^ In this work, we process the two steps in the GPU in a streamlined fashion without redundant data transfer between the host and the device. Moreover, the ANLM filters reported in Refs. 34 and 37 were designed to denoise MRI images, which feature a Rician noise. Although our reported work is primarily targeted to denoise Gaussian/Poisson noise in the MC outputs, we also added support for the Rician noise, so it can be readily used for processing MRI images.

### 2.2 GPU-accelerated Adaptive Nonlocal Means Algorithm and Optimizations

**Fig. 1:**
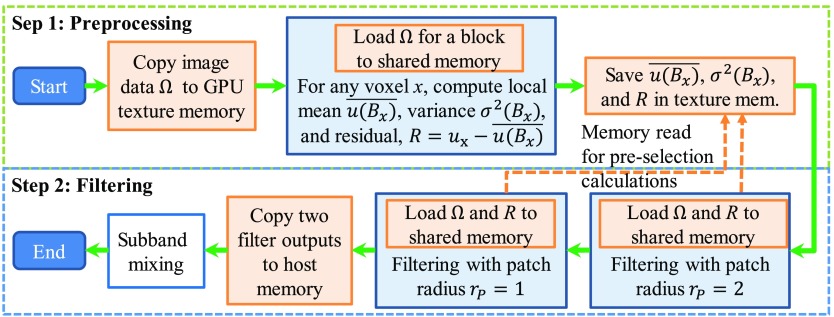
GPU ANLM filter algorithm workflow diagram. All memory operations are shaded in orange; the steps executed in the GPU are shaded in light-blue.

A workflow diagram for the developed GPU-accelerated ANLM filter is shown in Fig. 1. Overall, the GPU-ANLM filter is performed in two steps: (1) preprocessing: to compute R(x), $\bar{u(B_{x})}$, and the image variance within $B_{x}$, i.e., $\sigma^{2}(B_{x})$; the former two are needed in Eq. (3) and the latter two needed for patch preselection; (2) ANLM filtering: update image values according to Eqs. (1) and (2), respectively. In this work, both steps are implemented in parallel computing on the GPUs.

As demonstrated previously,^37^^,^^42^ caching of image data using the high-speed shared memory in the GPU is crucial for efficient GPU implementations. In this work, a number of extra optimization steps have been implemented over the work by Granata et al.^37^ to further improve the computational efficiency. These improvements include: (1) loading the preprocessing outputs, R(x), to the shared memory at the beginning of step 2 (referred to as “O1”), (2) using 3-D blocks instead of 2-D blocks^37^ to maximize cache reusability between threads (referred to as “O2”), (3) precomputing R(x), $\bar{u(B_{x})}$, and $\sigma^{2}(B_{x})$ using the GPU instead of a CPU (referred to as “O3”), and (4) streamlining the two filtering steps for wavelet sub-band mixing^34^ on the GPU without redundant data transfer (referred to as “O4”).

**Fig. 2:**
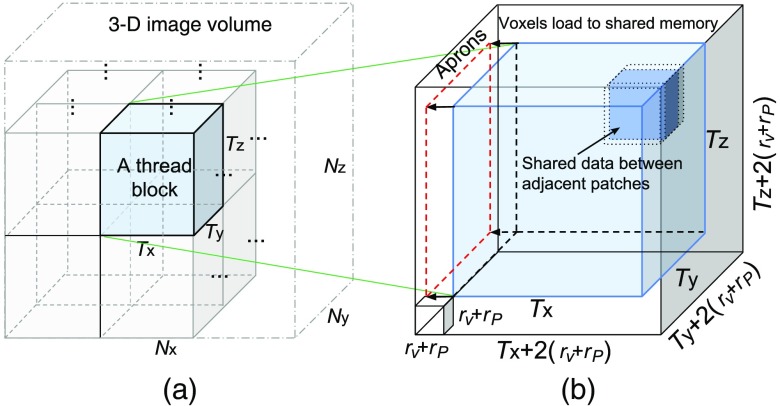
GPU thread and memory mapping: (a) image-to-thread space mapping and (b) image subvolumes loaded to the shared memory. In (a), an $N_{x}\times N_{y}\times N_{z}$ image volume is partitioned into blocks of size $T_{x}\times T_{y}\times T_{z}$, with each block filtered by a thread block^43^ of the same size and each voxel in the block updated by a single thread. In (b), filtering a single block requires to load not only the block-mapped voxels (light-blue) but also the voxels within a margin, referred to as the “aprons,” determined by the radii of the patch ($r_{P}$) and search area ($r_{V}$).

The partition of the raw image volume to the GPU thread/block space and mapping to the shared memory are shown in Fig. 2. Here we want to highlight the benefit of moving from a 2-D thread block^37^ to a 3-D thread block design. For example, to filter a total of 4096 voxels using a filter of $r_{V}=3$, $r_{P}=2$, an isotropic 3-D block configuration of $T_{3D}^{3}$ threads, where $T_{3D}=T_{x}=T_{y}=T_{z}=4096^{1/3}=16$ requires reading data from a total of ${\left[T_{3D}+2(r_{V}+r_{P})\right]}^{3}=17,576$ voxels. Specifically, $2(r_{V}+r_{P})$ here represents the additional layers of the marginal voxels, in each dimension, that are to be loaded into the shared memory, referred to as “aprons” in Fig. 2(b), along with the voxels needed for the filtering calculation. If one uses an isotropic 2-D block configuration of $T_{2D}^{2}$ threads, where $T_{2D}=\sqrt{4096}=64$, this operation needs data from ${\left[T_{2D}+2(r_{V}+r_{P})\right]}^{2}\times [1+2(r_{V}+r_{P})]=60,236$, about 3.5× the size of the 3-D block case. Thus, the 3-D thread block design can effectively reduce the shared memory usage by reducing the apron size.

### 2.3 Metrics

We use the signal-to-noise ratio (SNR) to evaluate the image quality improvement due to the denoising filter. Please note that the MC simulation noise is spatially and photon-count dependent. The SNR can be calculated by running multiple (here we use N=1000) independently seeded MC simulations, each with K photons, and computing the mean, $\mu_{K}(r)$, and standard deviation, $\sigma_{K}(r)$, at any given voxel located at r, then converting to dB as

$${\mathrm{SNR}}_{K}(r)=20\;\log_{10}\frac{\mu_{K}(r)}{\sigma_{K}(r)}.$$ (4)

The average SNR difference before and after applying the denoising filter, $\Delta {\mathrm{SNR}}_{\mathrm{filter}}$, is subsequently calculated along selecting regions-of-interest for various photon numbers and heterogeneity settings.

On the other hand, if we assume the MC noise has a shot-noise-like behavior, by increasing photon numbers from N to c×N, where c>1, we can anticipate an overall dB SNR improvement of $\Delta \mathrm{SNR}=20\;\log_{10}\sqrt{\frac{c\times N}{N}}=10\;\log_{10}c$. For example, a 10-fold increase in photon number in an MC simulation brings a roughly 10-dB SNR improvement. Once ${\Delta \mathrm{SNR}}_{\mathrm{filter}}$ is calculated, the above equation allows us to estimate a filter-equivalent photon number multiplier $M_{F}$ as

$$M_{F}=10^{{\Delta \mathrm{SNR}}_{\mathrm{filter}}/10}.$$ (5)

The larger the $M_{F}$ value, the better the performance of the filter.

Furthermore, when assessing improvements due to various optimization strategies proposed in Sec. 2.2, we incrementally add the optimization steps and calculate the average runtime after each addition. To compare the speed improvement from the CPU-to-GPU-based ANLM filters, we also run multiple (N=3) independent trials for each simulation setting and calculate the average runtime differences. Although it is desirable to compare the GPU-ANLM reported in this work with the one published previously,^37^ this prior work does not contain the full ANLM implementation^34^ (see Table 1). In this case, when an optimization strategy involves a feature not available in Ref. 37, we fall back to the original ANLM algorithm^34^ to make the comparison.

## 3 Results

To evaluate the improvement of MC image quality using the denoising filter described above, a cubic domain of 100×100×100 grid with $1\;{\mathrm{mm}}^{3}$ isotropic voxels is used. Three benchmarks are tested: (B1) a homogeneous domain with absorption coefficient $\mu_{a}=0.02/\mathrm{mm}$, scattering coefficient $\mu_{s}=10/\mathrm{mm}$, anisotropy g=0.9, and refractive index n=1.37, (B2) same as B1, with the addition of a $40\times 40\times 40\;{\mathrm{mm}}^{3}$ cubic absorber centered at (50, 50, and 30) mm with 5× absorption contrast, i.e., $\mu_{a}=0.1/\mathrm{mm}$, and (B3) same as B2, but featuring a cubic inclusion of fivefold refractive index contrast instead of absorption, i.e., n=6.85. The last benchmark was designed to test for the edge-preservation capability of this filter.

In all benchmarks, a pencil beam pointing toward the +z-axis is located at (50, 50, and 0) mm. Photon counts ranging from $10^{5}$ to $10^{8}$, with 10-fold increments, are simulated. For each photon count, 1000 independently seeded simulations are performed. For the ANLM filter, the patch radii $r_{P}$ for the two independent filtering processes for wavelet sub-band mixing^34^ are 1 and 2, respectively. The box-filter domain radius $r_{B}$ is set to 1 as used in Ref. 34. The search radius $r_{V}$ is set to 3, resulting in a total search volume of ${\left(2r_{V}+1\right)}^{3}=343$ voxels. We also tested a larger search radius $r_{V}=5$ (not shown) and observed only a marginal SNR improvement. For better computational efficiency, $r_{V}=3$ is used in all examples here. The MC simulation was performed using Monte Carlo eXtreme (MCX).^13^ Two GPUs—NVDIA GTX 980Ti and 1080Ti—are used to test both the MC simulation and the ANLM filtering. The CPU used for these simulations is an Intel i7-6700K.

**Fig. 3:**
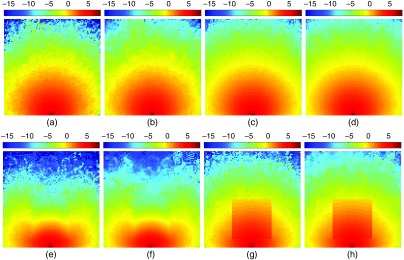
Comparisons between fluence cross-sectional plots before and after denoising in three benchmarks: (c and d) a homogeneous domain (B1), (e and f) a cubic domain with an absorbing inclusion (B2) or (g and h) a reflective inclusion (B3). In each pair, we show the fluence maps before (c, e, and g) and after (d, f, and h) the denoising filter. For visual comparison, we also show the plots for running $10^{6}$ photons for the homogeneous case in (a) and its denoised version in (b). All fluence plots are shown in $\log_{10}$ scale.

In Figs. 3(a)–3(d), we show the MC output images of benchmark B1 (homogeneous) at two selected photon counts—$10^{6}$ and $10^{7}$. The raw continuous-wave (CW) MC fluence outputs along the plane y=50  mm are shown in Figs. 3(a) and 3(c) for the two photon counts, and their corresponding denoised versions are shown in Figs. 3(b) and 3(d), respectively. Similarly, in Figs. 3(e)–3(h), the cross sections along the same plane (y=50  mm) for the absorbing inclusion (B2) and the refractive inclusion (B3) cases, respectively, are reported before and after denoising for simulations with $10^{7}$ photons.

**Fig. 4:**
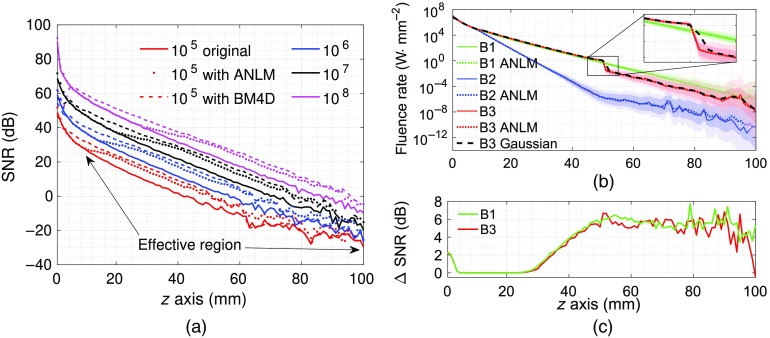
Comparisons of (a) SNR in dB, (b) fluence mean values before and after denoising at various photon numbers and domains, and (c) SNR improvement after ANLM filtering at $10^{8}$ photons. In (a), both the GPU-ANLM filter (dotted) and a CPU-BM4D^35^ (dashed) are tested in a homogeneous domain (B1). In (b), three benchmarks (B1—homogeneous, B2—absorbing inclusion, and B3—refractive inclusion) are processed; light and dark shaded regions represent one standard deviation from the mean before and after filtering, respectively. A Gaussian filter is applied to B3 and the cross section is shown in black dashed line. A zoom-in view of the inclusion edge in B3 is shown in the inset. All plots are extracted from the vertical line at x=50  mm and y=50  mm after running 1000 repeated simulations.

To quantitatively assess the image quality improvement, in Fig. 4(a), we show the SNR (in dB) profiles at various photon numbers before and after filtering along the midline of Fig. 3. Only the homogeneous domain (Benchmark B1) is shown here as a representative example. To assess the potential bias imposed by the denoising filter, we also plot the mean values of the fluence in Fig. 4(b). In this case, the mean fluence profiles for all three benchmarks (B1—green, B2—blue, and B3—red) are compared with a sample simulation at a photon count of $10^{6}$. The light and dark color-shaded regions represent the variations of the fluence within one standard deviation (calculated from 1000 repeated simulations) before and after filtering, respectively. To demonstrate the edge-smoothing effect of the conventional Gaussian filter, we also show the mean fluence along the same cross section for the refractive inclusion (B3) case after a 5×5×5 Gaussian filter with σ=0.67  mm. In addition, we found that the SNR improvement due to filtering is not strongly correlated to the presence of heterogeneities. As shown in Fig. 4(c), the SNR difference inside the refractive inclusion (B3) is similar to those in the background region, as well as in the homogeneous case (B1), despite the differences in fluence intensity.

**Fig. 5:**
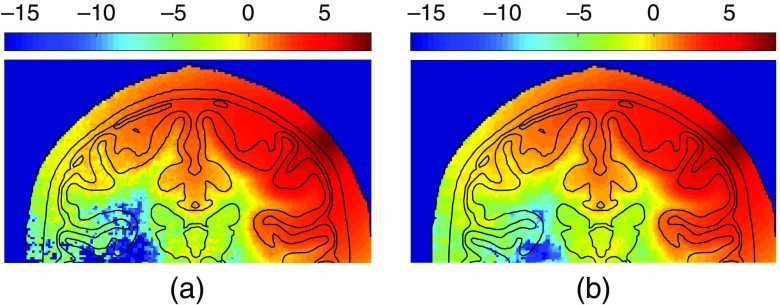
Comparisons between the simulated fluence images with $10^{8}$ photons, in coronal views, in a 19.5-year-old brain atlas (a) before and (b) after denoising. The fluence is shown in $\log_{10}$ scale.

To demonstrate the application of our GPU-ANLM filter in more complex tissue structures, a 19.5-year-old brain atlas with a voxel-domain size of 166×209×223 and $1\;{\mathrm{mm}}^{3}$ voxels is simulated.^44^ The atlas is segmented into five layers: scalp/skull, CSF, gray matter, and white matter. The optical properties for scalp/skull and CSF are based on literature,^6^ similarly for gray and white matters.^45^ A pencil beam is placed on the scalp with source direction perpendicular to the head surface. A total of $10^{8}$ photons are simulated. An ANLM filter of the same parameters as the above benchmarks is applied. In Fig. 5, we show the coronal section of the MC fluence output before and after the ANLM filtering.

**Fig. 6:**
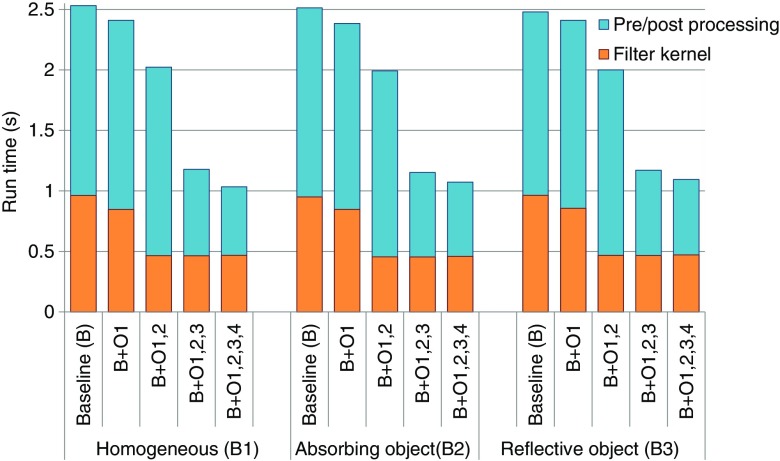
Filtering (orange) and pre-/postprocessing (blue) runtime comparisons when incrementally applying four optimization strategies in three benchmarks using $10^{7}$ photons: B—baseline, O1—using shared memory, O2—using 3-D blocks, O3—GPU-based preprocessing, and O4—streamlined two-step filtering.

Next, we investigate the computational speed improvements by incrementally incorporating the optimization strategies outlined in Sec. 2.2. All simulation runtimes are divided into two parts—the total pre-/postprocessing runtimes and the GPU ANLM filter kernel runtimes. In Fig. 6, the runtime comparison is reported as a stack-bar plot for the four different optimization methods and three benchmarks. As a reference, we designed a baseline (B) simulation by combining the features from the previously published CPU and GPU ANLM filters: we use the previously reported filter settings^37^ if it is implemented; otherwise, we use the settings from the CPU-ANLM filter.^34^ Features not described in the former include the nonlocal patch preselection and the wavelet sub-band mixing. In Fig. 6, the blue bars represent cumulative runtimes for the pre- and postprocessing steps; the orange bars denote the total runtimes of the ANLM GPU kernel. For this particular comparison, we use a photon number of $10^{7}$. The reported runtimes are averaged results from three runs.

## 4 Discussions and Conclusion

From visual inspection of Fig. 3, we found that application of the proposed GPU ANLM filter results in noticeable improvement in the smoothness of the MC fluence images. From comparing Figs. 3(a)–3(c), the denoised image in (b) shows quality improvement similar to that due to increased photon counts as shown in (c). A similar improvement can also be found between (d) and (c). We also found that image smoothness improvements in regions near the source are not as significant as those distal to the source. This is indicative of the adaptiveness of the filter—the denoising filter smoothens the image less in regions with high intensity (i.e., high SNR) than in regions of relatively high levels of noise.

Additional findings can be concluded from interpreting Figs. 3(e)–3(h). For both of the tested heterogeneous domains—one with an absorbing inclusion, shown in (e) and (f), and the other one with a refractive inclusion, shown in (g) and (h), the denoised images also demonstrate a noticeable improvement in overall image smoothness. Despite the overall smoothed contours, the image features, due to the inclusions, are well preserved when comparing the images from before and after filtering. The images in Figs. 3(g) and 3(h) are particularly notable because the sharp edges, a result of the discontinuity of fluence due to refractive index mismatch, show little sign of blurriness after the denoising.

From Fig. 4(a), it is clear that the ANLM filter (dotted lines) helps improve the SNR in all tested photon counts. However, it appears that such improvements are limited to regions distal from the source region, labeled as the “effective region” in Fig. 4(a)—the higher the photon count, the further the distance between the effective region from the source. This finding has mixed implications. On the positive side, it confirms the adaptiveness of the ANLM filter, as observed above, and ensures that the regions with (relatively) high SNRs receive minimal distortions. On the other hand, one can also anticipate that the effectiveness of the adaptive filter gradually reduces when processing MC outputs from increasing numbers of photons. This behavior is clearly different from running 10-fold more photons (solid lines), where the SNR enhancement appears to be relatively uniform. The tested CPU-BM4D filter (dashed lines) shows a wider effective region compared to ANLM except near the source, despite a much slower speed (20 s total run-time on a CPU) due to the lack of GPU implementation. The average SNR improvement in the BM4D algorithm is also slightly higher than that of ANLM. While we demonstrate significant improvements in MC photon simulation via denoising with the ANLM filter, it is not our intent to claim it is optimal for this task. Based on this figure, exploring and accelerating other contemporary 3-D adaptive filters, such as BM4D, could be promising future directions for this research.

To quantify the improvement in SNR due to the denoising filter, we heuristically determined the effective region boundary using an SNR improvement threshold, above which the improvement is considered. In addition, to minimize the bias due to the inaccurate SNR values in the low-photon region (for example, SNR<0  dB), we use median instead of mean to calculate the average improvement.^46^ The median SNR improvement in the entire volume ranges from 4.5 to 5.4 dB for various photon counts; this improvement increases to 5.2 to 5.5 dB if we only consider the effective regions where ΔSNR>3  dB. According to Eq. (5), such SNR improvements are equivalent to those produced by increasing the photon number by about 3.5-fold (i.e., $M_{F}=3.5$). We also estimated the median dB increments from $10^{5}$ to $10^{6}$, $10^{6}$ to $10^{7}$, and $10^{7}$ to $10^{8}$, which are 8.7, 9.6, and 9.8 dB, respectively. These results are close to the 10 dB increase as expected for shot-noise. Similarly, we estimated that the BM4D filter has a median 6.8 to 7.4 dB increase in SNR in regions where ΔSNR>3  dB, which yields an $M_{F}$ around 5.

The averaged fluence profiles after denoising align well with those of raw MC outputs, as shown in Fig. 4(b), in both homogeneous and heterogeneous cases. This confirms that the proposed denoising filter does not add noticeable bias to the data. Particularly, the sharp fluence change near the boundary of the refractive inclusion is well preserved after ANLM filtering according to Fig. 4(b) (red-lines). The efficacy of the denoising filter can also be seen from the notable shrinkage of signal variations from the light- and dark-shaded areas, particularly in regions far away from the source. The ANLM filter gives a significantly better result than the 3-D Gaussian filter, which tends to smooth all sharp features, as seen from Fig. 4(b) and its inset. Similar improvement can be found in Fig. 5 when using a complex head model. From Fig. 4(c), it also appears that the denoising SNR improvement is not strongly influenced by either the fluence magnitude or the background optical property variations. This allows us to extrapolate these assessments to more complex domains.

From Fig. 6, the total runtimes of the filtering step were reduced from 2.5 s to around 1 s—a nearly 2.5× speedup. On average, the filtering runtimes (orange) speedup because utilization of shared memory (O1) is around 11%; the addition of the 3-D block configuration (O2) further reduces the GPU filtering kernel runtimes by 50% from the baseline. Furthermore, by moving the preprocessing step from the CPU to the GPU (O3), we cut the preprocessing runtimes (blue) by 53%. Finally, the use of streamlined GPU-based wavelet sub-band mixing (O4) further reduces the postprocessing time by another 13%, yielding a nearly 60% total time reduction. The overall runtimes, as well as the speedup ratios due to various optimization strategies appear to be independent of the types of the inclusions in the media.

Two major observations can be made from the runtime data in Table 2. First, by comparing the runtimes between the CPU-based ANLM filter obtained from Refs. 34 and 47 and our GPU-based ANLM filter, we can observe a threefold to fourfold improvement in speed, with slight variations across different media configurations and photon numbers. Second, the relatively constant filtering runtimes, when combined with the proportionally increasing GPU MC runtime with respect to increasing photon numbers, suggest that the overall efficiency of combining an MC simulation with a denoising process depends on the simulated photon numbers—the larger the photon number, the greater the overall improvement in speed. According to our above discussions regarding Fig. 4(a), the denoising filter produces an SNR improvement similar to that of running a simulation with 3.5-fold photons. By multiplying the MCX runtimes by a factor of 3.5 and comparing the results with the summation of MCX and GPU-ANLM filter runtimes, we can conclude that the denoised MC simulation can reduce processing time in photon counts above $10^{7}$, with the maximum acceleration achieved at the highest photon counts. It is interesting to note that traditional CPU-based MC simulation has been known for slow computation.^8^ Combining the proposed denoising filter with the sequential MC simulation could have maximized the speed improvement (about 3.5×) in most of the photon counts if one continues to use CPUs for MC simulations. Highly parallel and efficient GPU MC codes, while being highly desired for biophotonics simulations, raise the thresholds for which this MC denoising method becomes effective. Despite that, according to Table 2, our proposed method could benefit a wide range of MC simulation settings, in particular, when combined with traditional MC simulations.

**Table 2:** The average total runtimes (in second) of the denoised MC simulations for different benchmarks and photon counts. Tests are performed using both NVIDIA 980Ti and 1080Ti GPUs. $T_{MC}$ and $T_{f}$ stand for the MC simulation and ANLM filtering runtimes, respectively.

Photon#	Runtime (s)														
	Homogeneous (B1)					Absorbing inclusion (B2)					Refractive inclusion (B3)				
	980Ti		1080Ti		CPU	980Ti		1080Ti		CPU	980Ti		1080Ti		CPU
	$T_{MC}$	$T_{f}$	$T_{MC}$	$T_{f}$	$T_{f}$	$T_{MC}$	$T_{f}$	$T_{MC}$	$T_{f}$	$T_{f}$	$T_{MC}$	$T_{f}$	$T_{MC}$	$T_{f}$	$T_{f}$
$10^{5}$	0.50	1.13	0.67	1.19	3.82	0.48	1.09	0.63	1.18	2.76	0.50	1.14	0.62	1.19	2.80
$10^{6}$	1.22	1.14	1.09	1.19	4.45	1.21	1.10	1.09	1.19	3.49	1.82	1.20	1.18	1.19	3.66
$10^{7}$	5.95	1.10	3.82	1.20	4.63	5.81	1.15	3.86	1.20	3.87	10.90	1.18	6.75	1.19	4.26
$10^{8}$	44.40	1.13	27.12	1.16	4.72	44.20	1.11	26.80	1.19	4.32	90.41	1.18	53.75	1.15	4.53

In summary, we reported a GPU-accelerated noise-adaptive nonlocal means (ANLM) filter to denoise low-photon MC simulation images and demonstrated an over 5-dB SNR improvement. This is equivalent to the SNR enhancement from running roughly 3.5 times more photons. The developed denoising filter shows excellent edge-preservation characteristics and low bias to the underlying fluence distribution, independent of photon numbers and the heterogeneities in the media. By developing a number of GPU optimization strategies, our GPU ANLM filter shows an overall 2.5-fold speed improvement from previous reported GPU-ANLM implementations, and a threefold to fourfold speed improvement from previously published CPU ANLM implementations. For a given domain size, we observed that the proposed denoised MC simulation gives the highest acceleration when the MC simulation runtime becomes dominant, such as at large photon counts or in media of high scattering coefficients. The reported whole-head denoising result also shows the promise of applying this technique to simulations in complex domains. With the support of both Gaussian and Rician noises, our GPU ANLM filter can also be broadly applied toward denoising other types of 3-D volumetric data including those from MRI scans. Both our GPU-MC simulation software (MCX^13^ and MCX-CL^15^) and the GPU-ANLM filter are open-source and can be downloaded from http://mcx.space/mcfilter/.
